# Which activity monitor to use? Validity, reproducibility and user friendliness of three activity monitors

**DOI:** 10.1186/1471-2458-14-749

**Published:** 2014-07-24

**Authors:** Brenda AJ Berendsen, Marike RC Hendriks, Kenneth Meijer, Guy Plasqui, Nicolaas C Schaper, Hans HCM Savelberg

**Affiliations:** Department of Human Movement Science, NUTRIM, School for Nutrition, Toxicology and Metabolism, Maastricht University Medical Centre, PO Box 616, Maastricht, 6200 MD the Netherlands; Department of Human Biology, NUTRIM, School for Nutrition, Toxicology and Metabolism, Maastricht University Medical Centre, PO Box 616, 6200 MD Maastricht, the Netherlands; Department of Internal Medicine, CAPHRI, School for Public Health and Primary Care, Maastricht University Medical Centre, Maastricht, the Netherlands

**Keywords:** Accelerometer, Wearing comfort, Posture classification, Sedentary, Feasibility, Reliability, Physical activity measurement

## Abstract

**Background:**

Health is associated with amount of daily physical activity. Recently, the identification of sedentary time as an independent factor, has gained interest. A valid and easy to use activity monitor is needed to objectively investigate the relationship between physical activity, sedentary time and health. We compared validity and reproducibility of physical activity measurement and posture identification of three activity monitors, as well as user friendliness.

**Methods:**

Healthy volunteers wore three activity monitors simultaneously: ActivPAL3, ActiGraphGT3X and CAM. Data were acquired under both controlled (n = 5) and free-living conditions (n = 9). The controlled laboratory measurement, that included standardized walking intensity and posture allocation, was performed twice. User friendliness was evaluated with a questionnaire. Posture classification was compared with direct observation (controlled measurement) and with diaries (free living). Accelerometer intensity accuracy was tested by correlations with walking speed. User friendliness was compared between activity monitors.

**Results:**

Reproducibility was at least substantial in all monitors. The difference between the two CAM measurements increased with walking intensity. Amount of correct posture classification by ActivPAL3 was 100.0% (kappa 0.98), 33.9% by ActiGraphGT3X (kappa 0.29) and 100.0% by CAM (kappa 0.99). Correlations between accelerometer intensity and walking speed were 0.98 for ActivPAL3, 1.00 for ActiGraphGT3X and 0.98 for CAM. ICCs between activity monitors and diary were 0.98 in ActivPAL3, 0.59 and 0.96 in ActiGraphGT3X and 0.98 in CAM. ActivPAL3 and ActiGraphGT3X had higher user friendliness scores than the CAM.

**Conclusions:**

The ActivPAL3 is valid, reproducible and user friendly. The posture classification by the ActiGraphGT3X is not valid, but reflection of walking intensity and user friendliness are good. The CAM is valid; however, reproducibility at higher walking intensity and user friendliness might cause problems. Further validity studies in free living are recommended.

## Background

Growing evidence shows the negative influence of both physical inactivity and sedentary behavior on health. It has been estimated that physical inactivity is currently related to 6% of mortality and is the main cause of 21-30% of several chronic diseases globally [1]. In addition, an Australian study suggested that 7% of deaths were attributable to prolonged sitting [2]. Recent studies suggest that an increase of physical activity could reduce metabolic risk independent of weight loss or aerobic fitness [3, 4]. In line with this, an increasing amount of evidence reveals an independent association between sedentary behavior and various health outcome measures [2, 5, 6]. However, the optimal amount, frequency and intensity of physical activity and the maximum amount and optimal distribution of sedentary time are still a matter of debate.

Reliable and valid measurements of physical activity and sedentary behavior are essential to draw sound conclusions about their influence on health. However, studies aimed at measuring sedentary behavior have often used self-reported data that suffer from subjectivity [7–9]. Both reproducibility and validity of self-report physical activity and sedentary behavior are variable [9, 10]. Accelerometry has been proposed as a method to objectively quantify sedentary behavior in addition to generally used measures of physical activity [11, 12]. Generally, accelerometers present counts per minute as an intensity outcome based on the accelerations. Previously, the counts per minute output has been tested and used to estimate sedentary time and activity [13, 14]. A problem of this approach is the inability to discriminate between sedentary time and standing time [15, 16]. Recently, several tri-axial activity monitors have been developed that enable measurement of posture (e.g. sedentary behavior and standing) by means of an inclinometer. The ActivPAL3™ (AP; PAL Technologies Ltd, Glasgow, UK), ActiGraphGT3X (AG; ActiGraph LLC, Pensalcola, FL, USA) and CAM (Maastricht Instruments BV, Maastricht, NL) are activity monitors which measure physical activity intensity, register time spent in different postures (e.g. lying, sitting and standing) and thereby assess sedentary time. The AP and the AG have often been used in epidemiological studies, whereas the CAM is a new device developed to provide raw acceleration data. Reproducibility and validity of this inclinometer function has rarely been studied. The posture classification by the CAM was validated in patients with chronic obstructive pulmonary disease and chronic heart failure in daily routine at home [17]. The inclinometer function of the AG showed limited validity and a dependence on location of application (hip vs. back) [13, 18]. Although several validation studies of the inclinometer function of the earlier manufactured uniaxial AP showed good posture classification [14, 15, 19–21], we are not aware of a study aimed at the validity of the posture classification function of the triaxial AP.

The validity and reliability of accelerometry measurements rely on wearing time [22]. However, the required hours per day and total days of measurement are not always met by all participants, which will lead to exclusion of data. Sufficient wearing comfort is a crucial factor in compliance and can consequently affect data quality and validity [23, 24]. Consequently, assessment of wearing comfort and attachment difficulty has been advised [17].

The aims of this study were to assess 1) reproducibility and validity of walking intensity and the posture classification of the AP, AG and CAM under laboratory conditions; 2) concurrent validity of the AP, AG and CAM with an activity diary in free living and 3) user friendliness of the three activity monitors.

## Methods

### Design

Data were acquired in both controlled and free-living measurements. In the laboratory measurement we compared data with observation, the gold standard; while the free-living measurements provided information in real daily life activities. In the laboratory measurement, the participants were instructed to follow a strict activity and posture protocol in a fixed setting. In the free-living measurement, participants were instructed to write down their activities in a diary every 15 minutes while wearing the devices in daily living. All participants completed a user friendliness questionnaire directly after the laboratory measurement or after returning the activity monitors when participating in the free-living measurement.

### Participants

A convenience sample of 14 healthy adults with normal BMI participated in the study. Five of them participated in the laboratory measurement (4 male, 1 female, mean age 22.4 years ± 2.2; mean BMI 22.3 ± 1.8); and nine participated in the free-living measurement (4 male, 5 female, mean age 27.2 years ± 8.3; mean BMI 21.3 ± 1.8). Informed consent from participants was obtained. This study was approved by the ethics committee of Maastricht University Medical Centre.

### Activity monitors

In this study we assessed three tri-axial activity monitors: the ActivPAL3 (AP); the ActiGraphGT3X (AG); and the CAM (Table 1). Both during the laboratory and in free-living measurements, participants wore all three activity monitors simultaneously. Wearing instructions were always provided by the main researcher.

**Table 1:** **Characteristics of the activity monitors and software**

	ActivPAL3	ActiGraph GT3X	CAM
**Size**	53 × 35 × 7 mm	38 × 37 × 18 mm	63 × 45 × 18 mm
**Weight**	15 g	27 g	100 g
**Placement**	Thigh	Waist	Thigh
**Application**	Adhesives	Elastic belt	Elastic belt
**Range**	2G	3G	4G
**Sample frequency**	20 Hz	30 Hz	25 Hz
**Waterproof**	Yes	No	No
Software*	ActivPAL software 6.0.2	Actilife 5.10.0	Custom Matlab program
**Classifications**	Sitting/lying	Lying	Sitting/lying
	Standing	Sitting	Standing
	Stepping	Upright	Active
**Intensity measure**	Metabolic Equivalent (MET)	Counts	Integrated Magnitude Area (IMA)
Epoch length*	1 s	1 s	1 s
Non-wear classification*	No	Yes (inclinometer code)	No

*Device offers more options; the option selected in this study is presented.

The AP was taped to the skin at the thigh, using double adhesive PALstickies™ in the laboratory measurement. In the free-living measurement, the AP was waterproofed and attached with a Tegaderm™ dressing (3 M Healthcare, St. Paul, MN, USA); and participants were instructed not to remove it for sleeping or showering. The AG was worn at the waist by means of an elastic belt and the participants were instructed to wear it at their back. As the AG is not waterproof, the device was to be removed when there was a risk of getting wet and during sleeping. To process the AG data, the ActiLife low frequency extension was used. The CAM was worn in an elastic belt around the thigh; also this device was to be removed during sleeping and when there was a risk of getting wet, because it is not waterproof.

The AP and CAM classify time as sitting/lying, standing and activity. The inclinometer function of the AG classifies time as sitting, lying and upright. For the analyses of the activity monitors individually, we assessed all classifications provided. In addition, we used sitting/lying time and upright time as generic measures in the laboratory measurement, to allow comparison between the three activity monitors. Sitting/lying time was defined as lying and sitting postures (regardless whether sitting time was misclassified as lying and vice versa by the AG inclinometer); and upright time was defined as all time spent in an upright orientation (regardless whether active time was misclassified as standing and vice versa by the AP and CAM). Besides the inclinometer function, the AG also discriminates between static posture (lying, sitting and standing) and activity based on a cut point of 100 counts on the vertical axis. For the AG only, the validity of this cut point was assessed in the free-living measurement.

### Laboratory measurement

**Figure 1:**
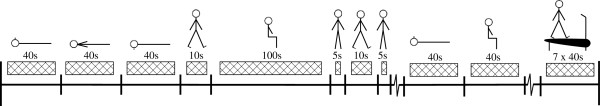
**Composition of postures and activities of the protocol execution.** Postures (lying, sitting and standing) and activities (walking over ground and on treadmill) are depicted with corresponding included time blocks.

In the laboratory measurement, we assessed the intensity measure and the inclinometer function to discriminate postures of the three activity monitors. To determine test-retest reproducibility, a protocol of 19.5 minutes was carried out twice by all participants, with a maximum of one day between measurements. The protocol consisted of periods of lying, sitting, standing, walking over ground and walking and running on the treadmill (Figure 1). Instructions were given orally. Four minutes were spent in a lying position, of which one minute on the side, one minute prone and two minutes supine. The protocol included two separate periods of sitting still on a chair. Thirty seconds were spent in a standing position (two periods of 15 seconds) and participants walked over ground two times. Finally, seven minutes were spent on the treadmill, walking with a speed of 0.3 m/s up to 3.0 m/s. Speed was increased with 0.3 m/s every minute up to 1.5 m/s, followed by 2.0 m/s and 3.0 m/s. In case of deviations from the protocol, time and nature of the deviations were registered and corresponding time periods were excluded from the analyses. The measurement included different posture allocations, leading to transition periods in the data in between the allocations. The devices were synchronized with the protocol and each other by means of jumping at the start of the measurement (CAM) or their internal clocks (AG and AP). To prevent inclusion of transition phases, the first and last ten seconds of the data of each condition were excluded; if the condition duration was 30 seconds or less, the first and last five seconds were excluded. In analyses, a total sitting/lying time of 300 seconds, a total standing time of 10 seconds, and a total time with walking over ground of 20 seconds were used of each laboratory measurement (Figure 1). For the AP, for each treadmill walking speed the average intensity was calculated with the middle 30 seconds to exclude transition phases, because intensity data per 15 seconds was used. For the AG and CAM the middle 40 seconds of each treadmill walking speed were used to calculate average intensity.

### Free-living measurement

We evaluated four methods in the free living experiment (AP, AG inclinometer, AG counts and CAM). During the free-living measurement, participants wore the three activity monitors simultaneously for at least 3 days. All activity monitors were set to measure 24 hours per day. Participants filled out an activity diary every 15 minutes from waking up till going to bed, writing down the amount of minutes spent in four categories: sitting, walking, standing and other activities. These four categories were then classified as sitting/lying, standing and active. When activities occurred in only one category for longer than 15 minutes, participants were allowed to report them after the subsequent transition. Agreement with the diary was analysed per day. Minutes spent in each category were summed to a total day score. If the amount of minutes per hour registered in the diary exceeded or did not reach 60 minutes, minutes per category were normalised to match 60 minutes in total (referred to as corrected diary data). Both original and corrected diary data were used as comparator for the classification by the activity monitors in free living.

### User friendliness questionnaire

User friendliness was assessed in all participants with a self-administered questionnaire that was specifically developed for this study (Table 2). The questionnaire consisted of eleven Likert-scale questions for each activity monitor and asked about their preferred activity monitor and were all completed directly after the measurement. The questions are summarised into six categories: self-positioning and removal (Cronbach’s alpha 0.60), awareness of wearing (Cronbach’s alpha 0.86), limitations in behavior (Cronbach’s alpha 0.75), advice and embarrassment. In all categories, a high score represents high user friendliness.

**Table 2:** **The questions within each category of the user friendliness questionnaire**

Category	Question
**Self-positioning and removal**	1. The activity monitor is easy to apply/position
	2. The activity monitor is easy to remove
	3. The activity monitor is difficult to apply (recoded)
**Awareness of wearing**	4. The activity monitor fits easily underneath clothing
	5. I forgot I was wearing the activity monitor
	6. I noticed wearing the activity monitor while doing my daily activities (recoded)
**Limitations in behavior**	7. The activity monitor limits me during my daily activities (recoded)
	8. The activity monitor limits me when I’m exercising (recoded)
	9. I’ve changed my activity pattern because of the activity monitor (recoded)
**Advice**	10. I would recommend the activity monitor
**Embarrassment**	11. I would be ashamed if others would see I was wearing the activity monitor (recoded)

### Analyses

The reproducibility of posture classification during the laboratory measurement was analysed on a second-by-second basis with Cohen’s kappa for nominal data, for each activity monitor individually. A kappa-value of < 0.4 was defined as low agreement, > 0.4 was moderate, > 0.6 was substantial and > 0.8 was almost perfect agreement [25]. The reproducibility of the mean intensity of walking during the treadmill exercise was assessed with Intra Class Correlation (ICC) and Bland Altman plots.

Observation was used as gold standard in the laboratory measurement. Data from both laboratory measurements were pooled for validity analyses. Percentages of correctly classified seconds by each activity monitor were calculated and Cohen’s kappa was used to test agreement with the protocol on a second-by-second basis. Friedman’s ANOVA assessed whether the percentages of correctly classified sitting/lying and upright time differed between the three activity monitors. Correlations between walking speed and mean intensity per participant as provided by the standard software were calculated. Concurrent validity between posture classification by the activity monitors in the free-living measurements and the diaries was assessed with ICC and Bland Altman plots. The CAM and AG were only worn during wake time; therefore, their analyses were performed on wake time diary data.

Differences in the category scores of user friendliness between activity monitors were tested with Friedman’s ANOVA and Wilcoxon signed rank test (with an adjusted significance level of p < 0.0167). In addition, compliance in the free living measurement was registered.

Data were described as mean ± SD; if data was not distributed normally, median and 25^th^ and 75^th^ percentile were calculated. Statistical analyses were performed with SPSS version 19 and with a two-tailed significance level of 0.05 (unless mentioned differently).

## Results

### Laboratory measurements

**Figure 2:**
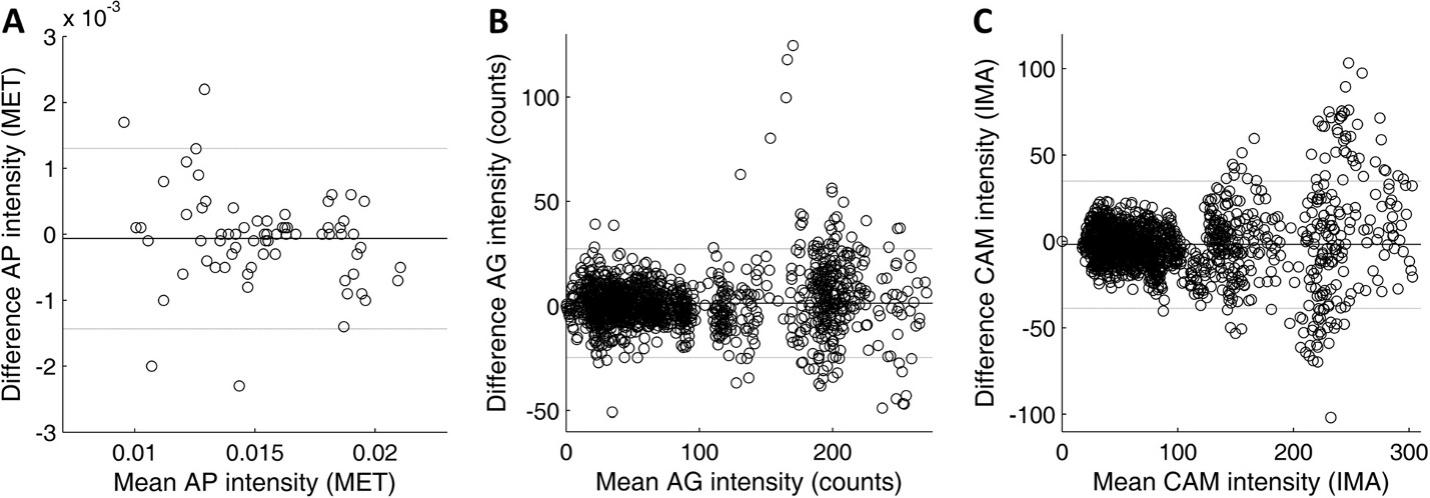
**a-c. Bland Altman plots for test and retest measurements of intensity, displayed for AP (A), AG (B) and CAM (C).** Black lines display mean difference and grey lines display mean difference ± 2 standard deviations. Mean difference and levels of agreement per activity monitor: AP -0.1 · 10^-3^ MET (-1.4 · 10^-3^ – 1.3 · 10^-3^), AG 1.4 counts (-24.6 – 27.3) and CAM -1.7 counts (-38.7 – 34.9).

Test-retest reproducibility kappas of the posture classification function were 0.99 in AP, 0.75 in AG and 0.95 in CAM (all p < 0.001). Kolmogorov-Smirnov tests showed that almost all intensity data during treadmill walking of the three activity monitors were not distributed normally; therefore, Spearman’s rho was used to assess test-retest reproducibility of the activity intensity during treadmill walking. The correlations between test and retest of the intensity measures were 0.97 in AP, 0.97 in AG and 0.96 in CAM (all p < 0.001). Evaluation of Bland Altman plots revealed no systematic differences in the two measurements for both AP and AG; however, the differences between the two measurements of the CAM increased with larger intensity (Figure 2a-c).

Percentages of correctly classified time were not distributed normally; therefore, median values and 25^th^ and 75^th^ percentile were calculated. Sitting/lying, standing and walking time were classified correctly by the AP in 100.0% of the time with a kappa-value of 0.98 (p < 0.001) and the same categories were correctly classified by the CAM in 100.0% of the time with a kappa-value of 0.99 (p < 0.001). Sitting, lying and upright time were classified correctly by the AG in 33.9% of the time with a kappa-value of 0.29 (p < 0.001) (specified for all categories and activity monitors in Table 3). Because of low correct posture identification, we looked at misclassification by AG in detail. In all participants a substantial amount of time spent lying was misclassified as non-wear by the AG (Table 4). In three participants the AG misclassified more than half of sitting time as upright. In one participant, sitting time was misclassified as both upright and non-wear. Overall 98.1% of classified non-wear occurred during lying, 1.7% occurred during sitting and 0.2% occurred during upright time.

**Table 3:** **Correct classification in the laboratory measurement for each category specifically**

	ActivPAL3	ActiGraphGT3X	CAM	P-value
**Sedentary**	100.0% (100.0-100.0)	35.7% (28.6-51.0%)	100.0% (99.5-100.0%)	0.010
**Sitting**	-	33.9% (0.0-83.2%)	-	
**Lying**	-	24.7% (22.5-32.2%)	-	
**Upright**	100.0% (100.0-100.0%)	96.7% (95.0-96.7%)	100.0% (100.0-100.0%)	0.007
**Standing**	100.0% (100.0-100.0%)	-	100.0% (90.0-100.0%)	
**Walking**	100.0% (100.0-100.0%)	-	100.0% (100.0-100.0%)	
**Cohen’s kappa**	.98*	.29*	.99*	

Percentages are depicted as median (25^th^ percentile – 75^th^ percentile); *p < .001.

**Table 4:** **Classification of sitting and lying time by the ActiGraphGT3X in percentages for each participant (1–5)**

			Classified as:			
Participant	Posture	Valid seconds	Lying	Sitting	Upright	Off (non-wear)
1	Lying	315	22.5%^#^	1.3%	1.9%	74.3%
	Sitting	280	0.0%	33.9%^#^	63.6%	2.5%
2	Lying	320	24.7%^#^	5.9%	2.8%	66.6%
	Sitting	280	0.0%	0.0%^#^	100.0%	0.0%
3	Lying	320	59.1%^#^	0.6%	3.1%	37.2%
	Sitting	215	0.0%	0.0%^#^	100.0%	0.0%
4	Lying	320	32.2%^#^	0.0%	5.9%	61.9%
	Sitting	265	0.0%	98.9%^#^	1.1%	0.0%
5	Lying	310	16.5%^#^	5.5%	3.9%	74.2%
	Sitting	280	0.0%	83.2%^#^	13.2%	3.6%

^#^Percentage correct classification.

Friedman ANOVAs showed that the ability to classify sitting/lying and upright time differed between the three activity monitors (sedentary: p = 0.010; upright time: p = 0.007), in which the AP and CAM performed similarly and the AG had a lower percentage of correct classification in both categories (Table 3).

The validity analyses of the intensity measures resulted in ICCs of respectively 0.98 (CI: 0.97 - 1.00), 1.00 (CI: 1.00 - 1.00) and 0.98 (CI: 0.97 - 1.00) between the treadmill walking speed and mean intensity measures of the AP, AG and CAM (all p < 0.001).

### Free-living measurements

**Figure 3:**
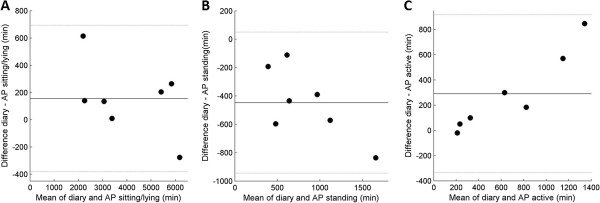
**a-c. Bland Altman plots of time registered sitting/lying (A), standing (B) and active (C) in diaries and time classified by AP.** Black lines display mean difference and grey lines display mean difference ± 2 standard deviations. Mean difference and levels of agreement in minutes per category: Sitting/lying 156.6 (-381.8 – 695.0), standing -447.1 (-944.5 – 50.2), active 290.7 (-336.4 – 917.8).

**Figure 4:**
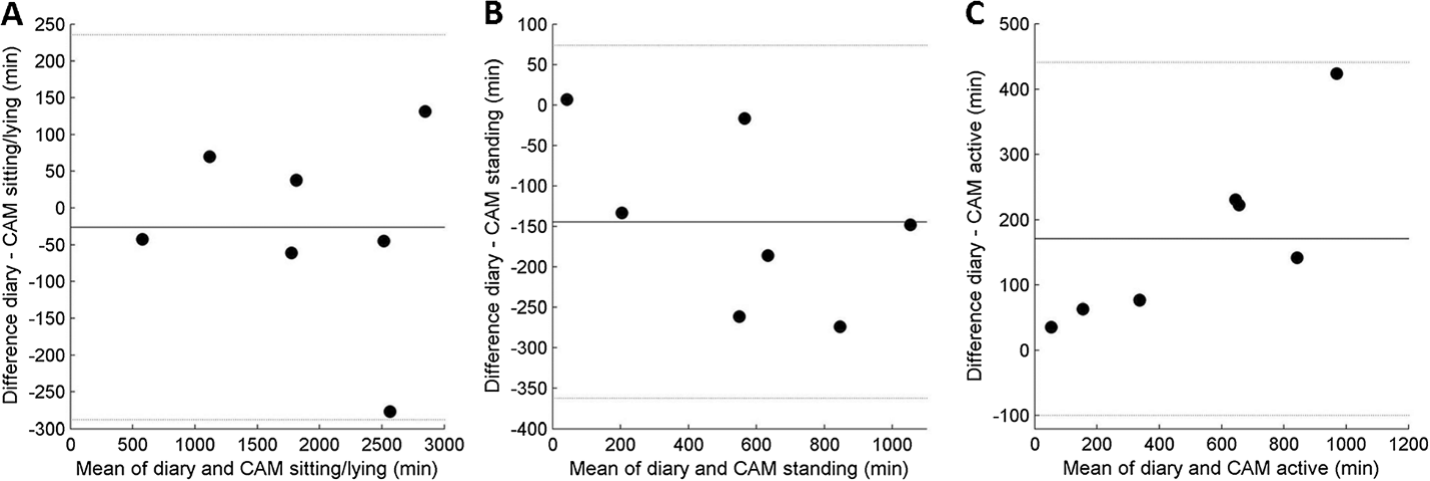
**a-c. Bland Altman plots of time registered sitting/lying (A), standing (B) and active (C) in diaries and time classified by CAM.** Black lines display mean difference and grey lines display mean difference ± 2 standard deviations. Mean difference and levels of agreement in minutes per category: Sitting/lying -26.5 (-288.3 – 235.4), standing -144.5 (-362.8 – 73.8) and active 171.0 (-99.8 – 441.7).

**Figure 5:**
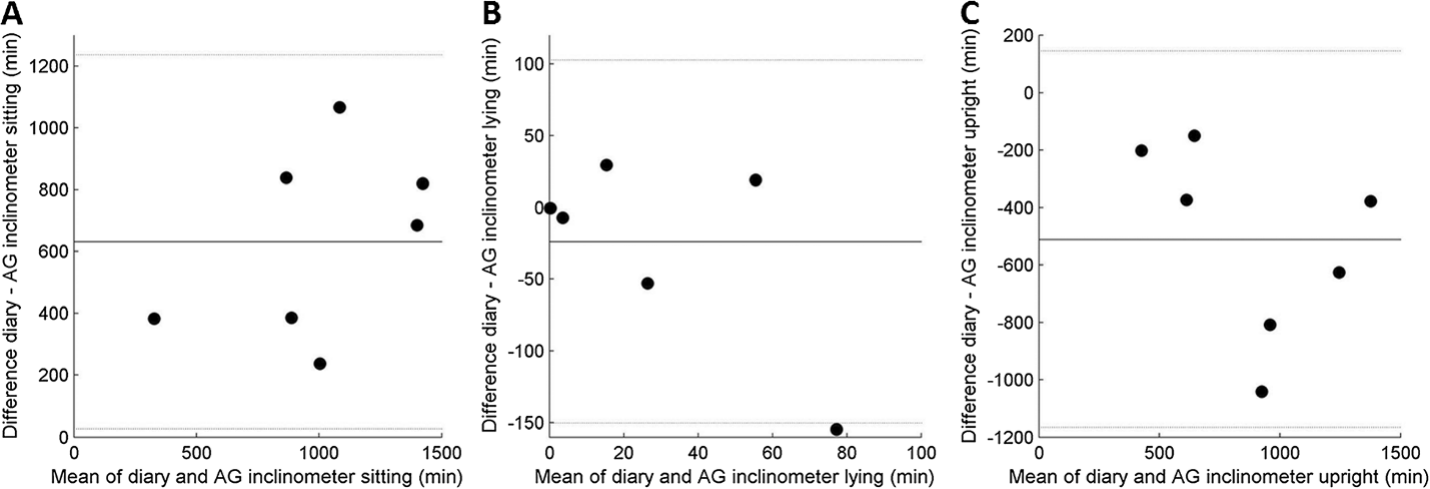
**a-c. Bland Altman plots of time registered sitting (A), lying (B) and upright (C) in diaries and classified by AG inclinometer.** Black lines display mean difference and grey lines display mean difference ± 2 standard deviations. Mean difference and levels of agreement in minutes per category: Sitting 631.0 (26.2 – 1235.8), lying -23.9 (-150.3 – 102.5) and upright -511.8 (-1167.2 – 143.6).

**Figure 6:**
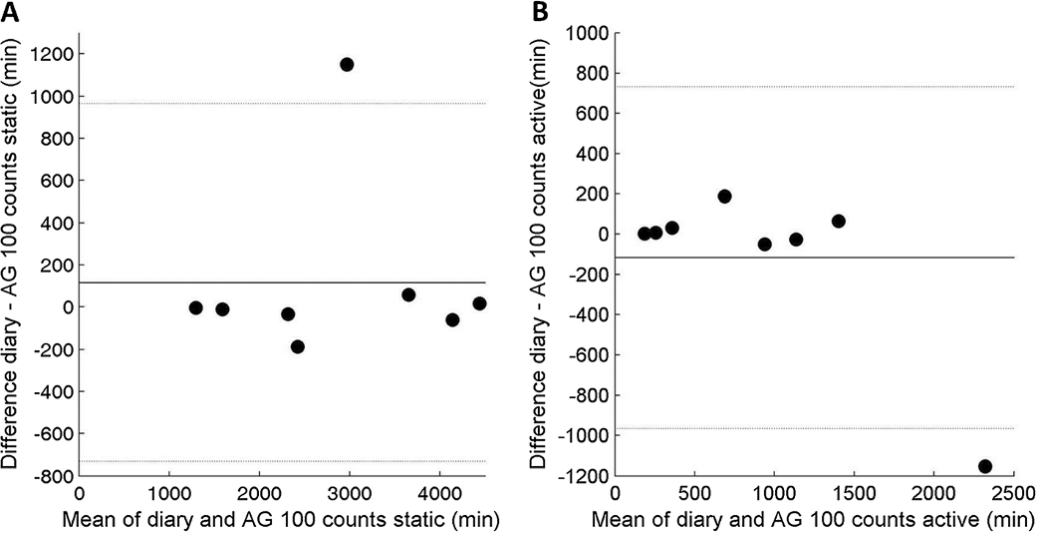
**a-b. Bland Altman plots of time registered static (A) and active (B) in diaries and classified by AG counts.** Black lines display mean difference and grey lines display mean difference ± 2 standard deviations. Mean difference and levels of agreement in minutes per category: Static 115.7 (-732.1 – 963.6) and active -116.7 (-966.1 – 732.7).

During the free-living measurements one participant did not wear the AP and AG and one other participant did not wear the CAM. All activity monitors were worn for a mean of four days, ranging from two to six days per participant (eight participants). On average per person, three days of the AP-data were usable (seven participants). An average of four days of the AG could be used for the 100 counts cut off point (eight participants) and an average of two days could be used for the inclinometer analyses (seven participants). An average of three days of the CAM measurements could be used in analyses (eight participants). Reasons for missing data were: the AP did not register data, the AG inclinometer did not register data, the CAM did not register data, the CAM stopped measuring before midnight and diary data was incomplete.To assess validity in free living, posture classification was compared with diary data. There appeared to be no difference in ICC-values between the comparisons with original diary data and with corrected diary data (to correct diaries not reaching 24 hours per day). ICC of the AP with the original and corrected diary outcomes was 0.98 (CI: 0.94 - 0.99). ICC of the CAM was 0.98 (CI: 0.95 – 0.99). Evaluation of Bland Altman plots revealed that according to AP and CAM, the total duration of activity was systematically lower and total duration of standing was systematically higher than registered in diaries (Figures 3 and 4). The ICC of the inclinometer function of the AG was 0.59 (CI: 0.22 – 0.81), upright time was systematically higher and sitting time was systematically lower than in the diaries (Figure 5). The distinction between static time and activity by the AG cut point of 100 counts had an ICC of 0.96 (CI: 0.88 – 0.98). The Bland Altman plots showed good agreement with diary, with exception of one participant in which static time was lower and active time was higher according to AG (Figure 6).

### User friendliness

**Figure 7:**
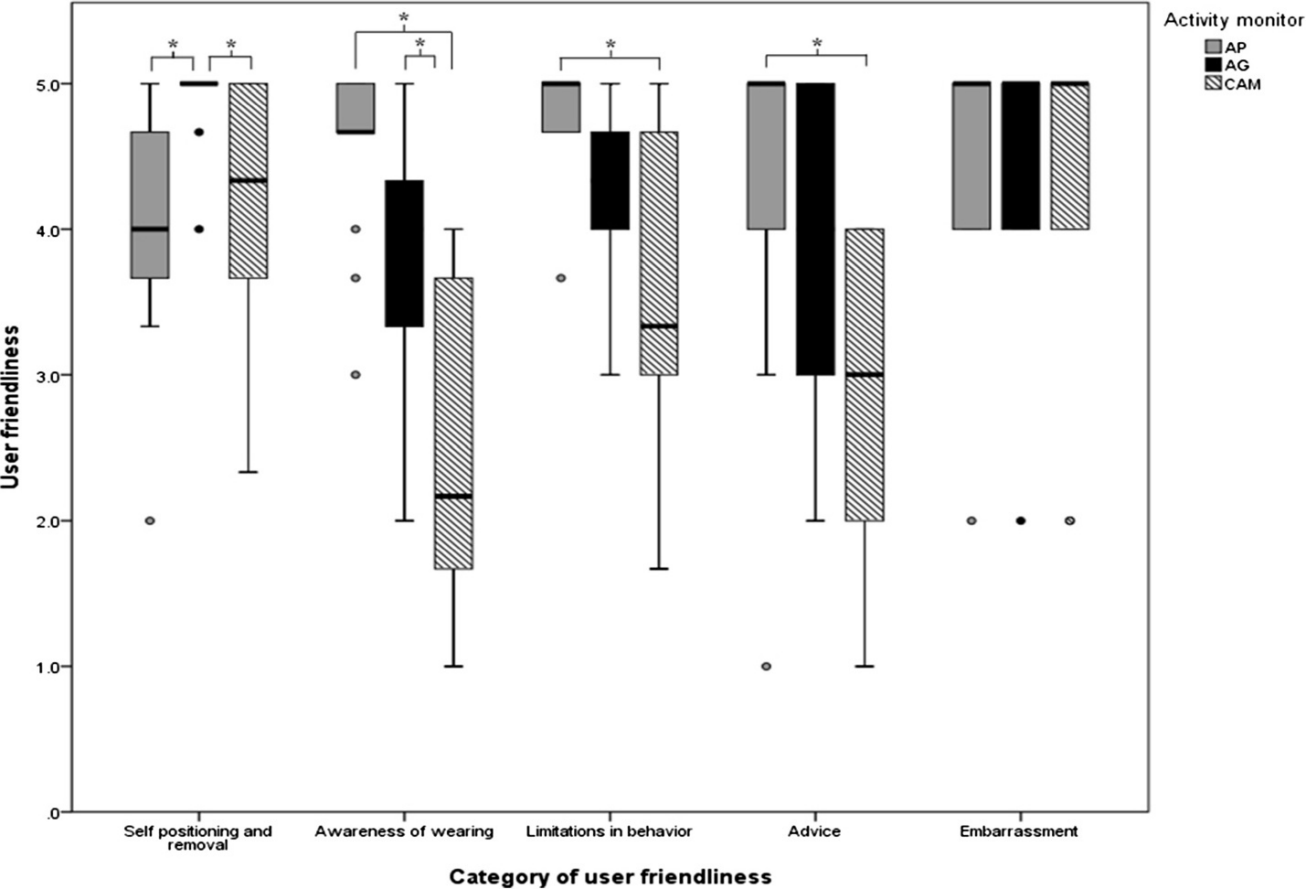
**User friendliness scores of the activity monitors for each category.** High scores represent high user friendliness. *Significantly different (p < 0.0167).

The activity monitors had significantly different scores on all question categories according to Friedman’s ANOVAs, except on the question regarding embarrassment to wear the devices (Figure 7). A Wilcoxon signed-rank test revealed that the AG had higher scores, i.e. was easier to use, with respect to self-positioning and removal than the CAM and AP (p = 0.011 and p = 0.003). On the awareness of wearing scale, the CAM scored significantly worse than the AG and AP, implying that participants were more conscious about wearing the CAM than wearing the other devices (p = 0.011 and p = 0.001). Participants experienced significantly more limitations in behavior with the CAM compared to the AP; the CAM also had significantly lower scores than AP with regards to advice (p = 0.008 and p = 0.007).

One participant of the laboratory measurement preferred the AP and four participants preferred the AG. Seven participants of the free-living measurements preferred the AP and two preferred the AG. None of the participants indicated CAM as preferred activity monitor to wear.

During the laboratory measurement one participant found it uncomfortable to remove the AP after a short period of measuring and two participants commented that the elastic belt of the CAM was uncomfortable. Following user friendliness issues occurred during the free-living measurements: reported skin irritation due to adhesive material of the AP (n = 3), AG was uncomfortable during sitting, lying or carrying a bag (n =5), skin irritation due to the elastic belt of the CAM (n = 2), aching muscles due to the elastic belt of the CAM (n = 1), CAM was uncomfortable due to sweating while playing sports, not fitting under clothes and did not stay in place (n = 3).

## Discussion

Choosing a suitable activity monitor for scientific studies depends on various aspects. This study aimed to address validity, reproducibility and user friendliness of three activity monitors available for measurement of physical activity and posture classification. Findings of our study indicate a trade-off between these three aspects in the AG and CAM. The AG shows moderate to high reproducibility but low validity for posture allocation and high user friendliness. The CAM shows moderate to high reproducibility, high validity, but low user friendliness. The AP scored well on all three aspects considered: high reproducibility, high validity and high user friendliness (despite reported skin irritation in four participants).

Both AP and CAM showed very good estimations of sitting/lying, standing and walking time. The postures were almost always classified correctly, indicating high validity. Other studies have shown this as well for CAM [17] and the uni-axial version of the AP [14, 15, 19, 20, 26]. The high reproducibility of the AP was in accordance with findings of a study aimed at the step counts of the uni-axial AP [27]. In the current study, reproducibility of the activity intensity estimated by the CAM at higher walking speed might be insufficient. This raises the question whether the CAM is able to adequately estimate activity intensity at higher intensities, a prerequisite for the discrimination of moderate and vigorous physical activity in pre-post measurements. Bearing in mind that the reproducibility analyses included data of only five participants, the fixation of the CAM by means of the elastic belt might not be secure enough and may have caused the low reproducibility at higher activity intensity.

The ICCs confidence intervals of the AP, AG counts and CAM were acceptable. However, the confidence interval of the ICC of the AG inclinometer function was wide, limiting generalizability to the population level. In addition, plots showed that differences with diary registration were large, despite the moderate to high ICC-values of classification by the activity monitors in daily living. The design of the free living part of the study refrains us from concluding whether the discrepancies were caused by misclassification of the devices or by inaccuracy of the diary as comparator. Participants were asked to report their activities every 15 minutes, as this was believed to be both feasible and accurate. Participants made an effort to report their daily activities in detail (i.e. in minutes precise). Nevertheless, reporting accuracy remains an issue which was not controlled for.

The AG inclinometer did not perform well in terms of reproducibility and validity of posture classification in both the lab and the free-living measurement. The second-by-second analysis of the laboratory measurement showed that much lying time is wrongly classified as non-wear by the inclinometer and sitting and upright time are often mingled. In addition, the amount and type of misclassification seems to be different between participants, for instance, in one participant 83.2% of sitting time was classified correctly, while sitting time in other participants was never classified correctly. The participants were instructed to wear the AG at the back during the measurements in this study because acceleration data reflects physical activity best when the device is worn at the lower back [28]. Although the AG manual states that the inclinometer function performs best when the AG is worn at the hip area, our findings are in line with the results of McMahon and colleagues who evaluated the validity of the inclinometer function when attached at back, waist and upper leg. The results of McMahon and colleagues indicated that compared to the waist, attachment to the back lead to more correctly classified standing time and less correctly classified sitting and lying time. Moreover, neither attachment location led to sufficiently correct sitting and lying identification [18]. Another study in which the AG was worn at the hip found correct posture classifications of 60.6% (standing) to 66.7% (lying). In that study, lying time, watching TV and sitting behind computer were also often classified as non-wear (respectively 14.3%, 6.5% and 9.3%). Also, watching TV and sitting behind computer were often classified as standing time (30.1% and 23.6%) [13]. Most remarkable is the amount of wrongly identified non-wear regardless of attachment location, especially in lying time. In our study, we adopted the non-wear classification provided by the inclinometer function. Usually, non-wear is identified with an algorithm based on a certain amount of inactivity [29–31]. These algorithms have been proven to be sufficiently valid to recognize non-wear in AG measurements [31]. Therefore, it might be advisable to reconsider the added value of the non-wear classification based on inclinometer data. In contrast to the inclinometer function, the discrimination of static and active time based on the cut point of 100 counts on the vertical axis was good. This is in agreement with previous studies [13, 14], which shows that when amount of activity is point of interest, regardless of sedentary time, the AG provides valid data.

Our user friendliness questionnaire addressed five aspects of which three have been proposed earlier. Application of activity monitors in free living requires a device that is easy to use, comfortable and unobtrusive [23, 24]. The CAM scores lowest in most subscales. Possibly, low scores decrease compliance and affect reflection of (in)activity patterns, due to obtrusiveness. The obtrusiveness of the CAM might be higher than the other two devices because of the relatively large size of the CAM and the large elastic belt that was used to wear it. However, compliance of participants to wear the activity monitors in this study was equal. This implies that the application method, removable (CAM and AG) or taped to the skin (AP), does not relate to compliance of wearing. Certain characteristics or subscales of user friendliness might be less or more important dependent on the goal and design of the study. In short measurements, the AG is preferred, whilst the AP is preferred in measurements of several days, even though skin irritation was reported by some participants. Further work is needed to relate the user friendliness to wearing compliance and behavioral adaptations.

The relative small sample size is a limitation of the current study. In addition, the sample consisted of only normal-weight, healthy adults. Therefore, results cannot be generalised to clinical or overweight adults and the user friendliness questionnaire should be assessed for validity and reproducibility in a larger, more variable population. Another limitation is the aforementioned lack of direct observation during free-living measurements. Machado-Rodrigues and colleagues showed that a more detailed diary yielded valid results against an accelerometer [32]. However, diaries always suffer from approximation and although participants were instructed to fill in their diary continuously, we could not control for recall bias in case of non-compliance. Therefore, it is not possible to draw solid conclusions about construct validity from these findings. Nevertheless, by including both controlled laboratory measurements and free-living measurements, our results give an indication of the reproducibility, validity and user friendliness of the three activity monitors.

## Conclusion

Results of activity monitoring depend on the device used, and choice of device should depend on the research aims and design. The majority of the studies which led to the current consensus on the negative influence of sedentary time on health, independent of physical activity, are based on subjective measures. As an objective measure, accelerometry can reinforce earlier results. The current study shows that the AP and CAM are able to classify posture and that the inclinometer function of the AG provides no valid posture classification. However, the AG can well be used if level of physical activity is of interest.
